# Predicting urine output after kidney transplantation: development and internal validation of a nomogram for clinical use

**DOI:** 10.1590/S1677-5538.IBJU.2018.0701

**Published:** 2019-07-27

**Authors:** Aderivaldo Cabral Dias, João Ricardo Alves, Pedro Rincon Cintra da Cruz, Viviane Brandão Bandeira de Mello Santana, Cassio Luis Zanettini Riccetto

**Affiliations:** 1 Unidade de Urologia e Transplante Renal, Instituto Hospital de Base do Distrito Federal (IHB), Brasília, DF, Brasil; 2 Divisão de Urologia, Faculdade de Ciências Médicas, Universidade Estadual de Campinas (UNICAMP), Campinas, SP, Brasil; 3 Unidade de Nefrologia e Transplante Renal, Instituto Hospital de Base do Distrito Federal (IHB), Brasília, DF, Brasil; 4 Divisão de Urologia, Hospital Universitário de Brasília (HUB), Brasília, DF, Brasil

**Keywords:** Kidney Transplantation, Nomograms, Delayed Graft Function

## Abstract

**Purpose:**

To analyze pre-transplantation and early postoperative factors affecting post-transplantation urine output and develop a predictive nomogram.

**Patients and Methods:**

Retrospective analysis of non-preemptive first transplanted adult patients between 2001-2016. The outcomes were hourly diuresis in mL/Kg in the 1^st^ (UO_1_) and 8^th^ (UO_8_) postoperative days (POD). Predictors for both UO_1_ and UO_8_ were cold ischemia time (CIT), patient and donor age and sex, HLA I and II compatibility, pre-transplantation duration of renal replacement therapy (RRT), cause of ESRD (ESRD) and immunosuppressive regimen. UO_8_ predictors also included UO_1_, 1^st^/0^th^ POD plasma creatinine concentration ratio (Cr_1/0_), and occurrence of acute cellular rejection (AR). Multivariable linear regression was employed to produce nomograms for UO_1_ and UO_8_.

**Results:**

Four hundred and seventy-three patients were included, mostly deceased donor kidneys’ recipients (361, 70.4%). CIT inversely correlated with UO_1_ and UO_8_ (Spearman's p=-0.43 and −0.37). CR_1/0_ inversely correlated with UO_8_ (p=-0.47). On multivariable analysis UO_1_ was mainly influenced by CIT, with additional influences of donor age and sex, HLA II matching and ESRD. UO_1_ was the strongest predictor of UO_8_, with significant influences of AR and ESRD.

**Conclusions:**

The predominant influence of CIT on UO_1_ rapidly wanes and is replaced by indicators of functional recovery (mainly UO_1_) and allograft's immunologic acceptance (AR absence). Mean absolute errors for nomograms were 0.08 mL/Kg h (UO_1_) and 0.05 mL/Kg h (UO_8_).

## INTRODUCTION

Many transplanted kidneys will not immediately function. One-fifth to one-third of deceased donor (1–4) and 3 to 5% of living-related allografts present either delayed (DGF) or slow graft function (SGF) (5, 6). Even when such outcomes are foreseen, because of longer cold ischemia times (CIT), poor quality of the allograft, patient age and co-morbidities, and immune sensitization (1, 7, 8), a sluggish functional recovery will increase monetary costs (9, 10) and lead to significant emotional strain. Moreover, the development of DGF likely shortens allograft survival (5, 11–16).

Allograft functional recovery is customarily assessed with serial plasma creatinine measurements (17). Yet, the most easily obtainable clinical parameter of allograft recovery is diuresis itself. Often the first question asked during clinical rounds addresses the patient's urine output, for an abundant and steady diuresis foreshadows timely functional recovery (18). Despite its clinical relevance, the current literature is void of predictive tools for post-transplantation diuresis, that should yield expected output according to the combination of the values of its predictor variables (19), and we gather that such tool could be used in the clinical environment to realistically manage patients’, and doctors’, expectations.

One should be reminded, however, that predictors’ effects can be nonlinear, and interpreting non-linear effects from complex multi-variable models through coefficients’ tables is no easy task. Such results are better digested when presented graphically; with, for instance, nomograms (20, 21). Notwithstanding its use as prediction tools, nomograms allow a more direct and intuitive understanding how each variable contributes to the outcome in complex models. We thus aimed to develop and internally validate, following Transparent Reporting of a Multivariate Prediction Model for Individual Prognosis or Diagnosis (TRIPOD) guidelines (22), a nomogram to predict urine output after kidney transplantation.

## PATIENTS AND METHODS

After Institutional Board Review, we retrospectively retrieved medical records from all consecutive patients older than 18 years who underwent non-preemptive deceased and living-related first kidney transplantation in our unit from January 2001 to January 2016. We excluded patients that died before the second postoperative day, those with severe urinary leakage - thus lacking a quantifiable urine output - as well as those with missing values for the outcome variables.

### Predictor variables

Continuous predictors included patient and donor age (years), duration of pre-transplantation renal replacement therapy (RRT, in years), cold ischemia time (CIT, in hours), last donor plasma creatinine concentration (donor creatinine, in mg %) and panel of reactive antibodies score (PRA in %, determined at most 6 months before transplantation). Ordinal predictors were Human Leucocyte Antigen mismatches in the A, B (HLA I) and DR (HLA II) loci. Categorical predictors included patient and donor sex, and End-Stage Renal Disease (ESRD) etiology, segregated into renal, systemic, urologic, autossomic dominant polycystic kidney disease (ADPKD) and undetermined causes. Additional categorical predictors were organ origin: whether the kidney came from live-related or from a deceased donor, whether due to vascular or non-vascular causes of brain death; initial immunosuppressive regimen: cycloporine and azathioprine (CSA+AZA), cyclosporine and mycophenolate (CSA+MMF), tacrolimus and azathioprine (FK+AZA), tacrolimus and mycophenolate (FK+MMF) and no use of calcineurin inhibitors either without (NoCalc) or with thymoglobulin (Thymo); and use of anti-interleucin 2 antibodies (anti-IL2: basiliximab or daclizumab) in initial immunossupression.

### Outcome Variables

**Figure 1:**
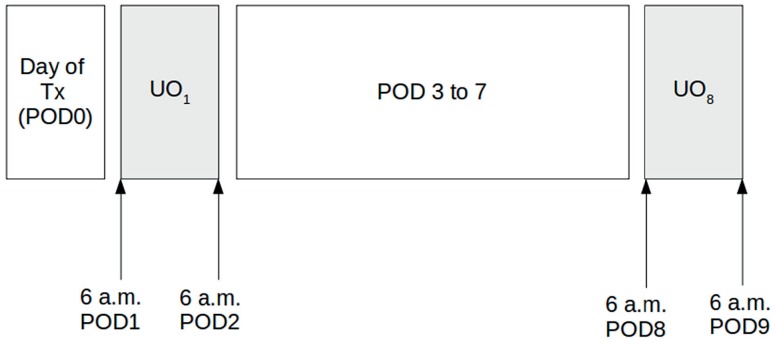
Visual representation of the intervals in which the response variable - urine output - was collected (light gray). Tx, transplantation, or postoperative day 0; UO1, urinary output from 6 a.m. of postoperative day 1 (POD1) to 6 a.m. of postoperative day 2 (POD2); UO_8_, urinary output from 6 a.m. of postoperative day 8 (POD8) to 6 a.m. of postoperative day 9 (POD9); POD 3 to 7, postoperative days 3 to 7.

Our outcome variables were hourly urine output in milliliters per patients's dry weight (mL/ Kg h), measured from 6 a.m. of the 1^st^ to 6 a.m. of the 2^rd^ postoperative day (UO_1_), where the day of the operation was considered postoperative day 0 (Figure-1), and hourly urine output in milliliters per patients's dry weight from 6 a.m. of the 8^th^ to 6 a.m. of the 9^th^ postoperative day (UO_8_). Regarding the latter outcome we added predictors from the initial postoperative course: Occurrence of biopsy-proven rejection episodes during the first postoperative week (AR), a categorical predictor; the ratio between 1^st^ and 0^th^ postoperative day plasma creatinine concentration (Cr_1/0_); and UO_1_, both the latter continuous predictors.

### Statistical analysis

#### Data description and bivariate analysis

We summarized continuous variables with medians and interquartile ranges (IQR), and proportions between categorical variables were displayed in frequency tables. Differences in urine output between categorical variables were assessed with Wilcoxon's and Kruskal-Wallis’ tests, the latter followed by Dunn's tests when appropriate. Spearman's rank correlation coefficients (*p*) were computed between continuous predictors and urine output.

#### Multivariable regression

All predictor variables were included in ordinary least squares linear regression models with UO_1_ and UO_8_ as response variables (henceforth, UO_1_ and UO_8_ models). Missing values were imputed with predictive mean matching (20, 23). Nonlinear predictors’ effects were modeled with 5-knot restricted cubic splines after investigation via Spearman's *p-p* ^2^ correlation (20). Redundant predictors were investigated with additive models, using an adjusted R^2^>0.90 cutoff. Model comparison was effected with the likelihood ratio test. Final models were validated and calibrated with 3000 bootstrap replicates. Analysis took place within the R statistical environment (24) augmented by the rms (25) and Hmisc (26) packages. P-values were computed where appropriate, with statistical significance established at 0.05.

## RESULTS

A total of 518 patients underwent kidney transplantation during the study period, and 45 cases were excluded: In 8 patients neither UO_1_ nor UO_8_ were recovered, 20 patients were younger than 18 years, 5 patients had undergone a second transplant and 7 underwent pre-emptive transplantation. Additionally, 3 patients died before the 1^st^ POD and 2 presented high output ureterocutaneous fistulae.

**Figure 2:**
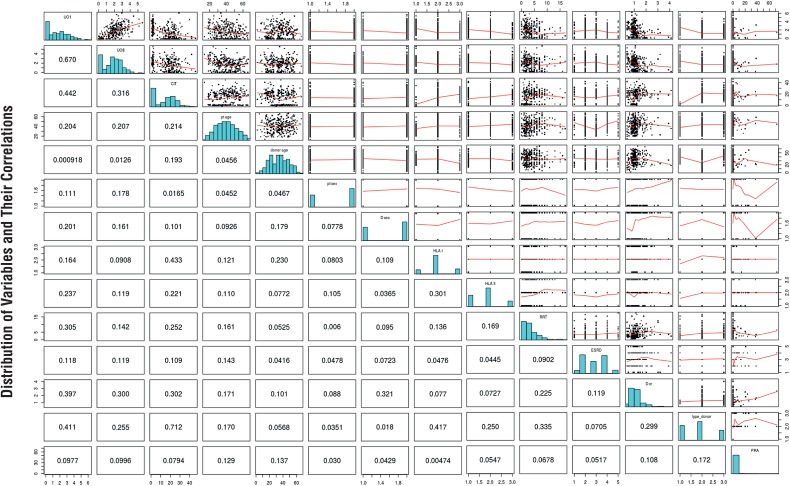
Matrix of pairwise scatterplots with *loess* regression lines (in red) and Spearman correlation coefficients (under the main diagonal). UO_1_, urinary output from 6 a.m. of postoperative day 1 (POD1) to 6 a.m. of postoperative day 2; UO_8_, urinary output from 6 a.m. of postoperative day 8 (POD8) to 6 a.m. of postoperative day 9; CIT, cold ischemia time in hours; pt age, patient age in years; donor age, age of donor in years; pt sex, patient sex; D sex, donor sex; HLA I, number of Class I HLA antigen mismatches (0, 3-1, 4); HLA II, number of Class II HLA antigen mismatches loci (0, 1, 2); dialysis, duration of renal replacement therapy before kidney transplantation; RRT, duration in years of renal replacement therapy before transplantation; ESRD, cause of ESRD aggregated in systemic, renal, urologic, autossomic dominant polycystic disease (ADPKD) and undetermined; D Cr, donor plasma creatinine in mg%; type of donor, whether the kidney came from a Deceased (vascular or non-vascular) or Living-Related donor; PRA, panel of reactive antibodies in %. Nomogram to predict UO1. In order to obtain the predicted hourly urinary output in mL/Kg, the user identifies each predictors’ values in their respective axes and uses a straightedge to approximate its score in the Points axis. All predictors values are added and this total score is identified in the Total Points axis. The predicted hourly urinary output can then be estimated in the UO1 mL/Kg h axis, also with a straightedge. This operation is considerably simplified with the use of a caliper. HLA DR, mismatches on the DR locus; IS, initial immunosuppression: csa_aza, cyclosporine + azathioprine; csa_mmf, cyclosporine + mycophenolate; fk_mmf, tacrolimus + mycophenolate; fk_aza, tacrolimus+azathioprine; thymo, thymoglobulin; no_calc_inhibitor, no calcineurin inhibitor.

Most remaining 473 patients were middle-aged (median 40 years), male (290/473, 61.3%), and received deceased donors’ allografts (321/473, 67.9%, supplemental Table-1). A third of the patients had either systemic (165/473, 34.9%) or undetermined (158/473, 33.4%, supplemental Table-2) ESRD etiology. Most donors were also male (262/453, 57.8%), which were younger than female donors (median 33.0 versus 39.0 years, p<0.001). Table-1 shows the distribution of categorical predictors and corresponding urine output (both UO_1_ and UO_8_), and Table-2 displays the distribution of continuous predictors and their correlations with UO_1_ and UO_8_. Figure-2 graphically displays the entire dataset along with pairwise Spearman's coefficients.

**Table 1:** Urine output at postoperative days 1 (UO_1_) and 8 (UO_8_) according to categorical predictors.

Variable	N (%)	UO_1_ (med, IQR)	P	UO_8_ (median, IQR)	P
**Patient sex**	Overall (473, 100%)	1.6 (0.3 – 2.8)	0.3 (W)	1.9 (1.0 – 2.6)	0.006 (W)
	Male (290, 61.1%)	1.5 (0.3 – 2.7)	–	1.8 (0.6 – 2.4)	–
	Female (183, 38.7%)	1.6 (0.5 – 3.0)	–	2.0 (1.2 – 2.7)	–
**Donor sex**	Overall (453, 95.8%)	1.6 (0.3 – 2.9)	0.001 (W)	1.9 (0.9 – 2.6)	0.07 (W)
	Male (262, 57.8%)	1.3 (0.2 – 2.6)	–	1.9 (1.2 – 2.7)	–
	Female (191,42.2%)	2.0 (0.6 – 3.1)	–	1.8 (0.7 – 2.5)	–
**Organ origin**	Overall (434, 91.8%)	1.6 (0.4 – 2.9)	<0.001^A^ (K)	1.9 (1.0 – 2.6)	<0.001A (K)
	Living-related (152, 35.0%)	2.6 (1.6 – 3.5)	–	2.2 (1.8 – 2.8)	–
	Non-vascular (176,40.6%)	1.2 (0.1 – 2.3)		1.6 (0.3 – 2.5)	
	Vascular (106, 24.4%)	0.6 (0.1 – 2.0)	–	1.4 (0.3 – 2.2)	–
**ESRD**	Overall (473, 100%)	1.6 (0.3 – 2.8)	0.18 (K)	1.9 (1.0 – 2.6)	0.004^B^ (K)
	Systemic (165, 34.9%)	1.4 (0.1 – 2.6)		1.6 (0.5 – 2.2)	
	Renal (104, 22.0%)	2.0 (0.3 – 3.0)		2.1 (1.1 – 2.8)	
	Urologic (24, 5.1%)	1.7 (0.6 – 2.8)		2.1 (1.3 – 3.3)	
	ADPKD (22, 4.7%)	1.2 (0.2 – 3.0)		1.2 (0.3 – 2.7)	
	Undetermined (158, 33.4%)	1.7 (0.6 – 3.0)		2.0 (1.4 – 2.6)	
**HLA I mm**	Overall (460, 97.3%)	1.5 (0.3 – 2.8)	<0.001^C^ (K)	1.9 (0.9 – 2.6)	<0.001^C^(K)
	4 (91,19.8%)	1.4 (0.2 – 2.6)		1.8 (0.5 – 2.5)	
	3-1 (310, 67.4%)	1.4 (0.3 – 2.7)		1.8 (0.7 – 2.5)	
	0 (59, 12.8%)	2.7 (1.5 – 3.3)		2.2 (1.6 – 2.9)	
**HLA II mm**	Overall (459, 97.0%)	1.5 (0.3 – 2.8)	<0.001^D^ (K)	1.9 (0.9 – 2.6)	<0.001^D^ (K)
	2 (69, 15%)	0.6 (0.1 – 2.1)		1.4 (0.2 – 2.2)	
	1 (254, 55.3%)	1.5 (0.4 – 2.7)		1.9 (0.9 – 2.6)	
	0 (136, 29.6%)	2.1 (0.9 – 3.1)		2.0 (1.3 – 2.7)	
**Initial IS**	Overall (473, 100%)	1.6 (0.3 – 2.8)	<0.001^E^(K)	1.9 (1.0 – 2.6)	<0.001^F^(K)
	FK+MMF (352, 53.5%)	1.2 (0.2 – 2.4)		1.7 (0.5 – 2.4)	
	CSA+MMF (90, 19.0%)	2.2 (1.0 – 3.2)		2.0 (1.1 – 2.8)	
	CSA+AZA (78, 16.5%)	2.4 (1.6 – 3.3)		2.2 (1.7 – 2.8)	
	FK+AZA (15, 3.2%)	2.5 (1.5 – 3.0)		2.5 (2.1 – 2.8)	
	Thymo (29, 6.1%)	0.2 (0.1 – 1.0)		0.8 (0.2 – 1.8)	
	No calc (8, 1.7%)	3.3 (2.5 – 4.1)		2.1 (1.8 – 3.0)	
**Anti-IL2**	Overall (473, 100%)	1.6 (0.3 – 2.8)	0.02 (W)	1.9 (1.0 – 2.6)	0.7 (W)
	Use (127, 26.8%)	1.2 (0.2 – 2.5)	–	1.7 (0.5 – 2.6)	–
	No use (346, 73.2%)	1.7 (0.5 – 2.9)	–	1.9 (1.2 – 2.6)	–
**AR**	Overall (473, 100%)	–	–	1.9 (1.0 – 2.6)	<0.001 (W)
	Yes (99, 20.9%)	–	–	1.3 (0.2 – 2.1)	–
	No (374, 79.1%)	–	–	1.9 (1.2 – 2.7)	–

A Significant differences in UO_1_ and UO_8_ between patients receiving allografts from deceased (vascular and non vascular causes) - versus living-related donors (P<0.001 for both comparisons);

B Significant differences in UO_8_ between patients with systemic and indeterminate ESRD causes (P=0.007);

C Significant differences in UO_1_ in patients with 0 versus 4 mismatches (P<0.001) and 3-1 versus 0 mismatches (P<0.001) and in UO_8_ in patients with 0 versus 4 mismatches (P<0.001) and 0 versus 3-1 mismatches (P<0.001);

D Significant differences in both UO_1_ in patients with 0 versus 2 mismatches (P<0.001) and in patients with 1 versus 2 mismatches (P=0.001) and in UO_8_ in patients with 0 versus 2 mismatches (P=0.001) and with 1 versus 2 mismatches (P=0.02);

E Significant differences in UO_1_ between patients receiving CSA+AZA versus FK+MMF (P<0.001), CSA+MMF versus FK+MMF (P=0.003), FK+MMF versus NoCalc (P=0.003), CSA+AZA versus Thymo (P<0.001), CSA+MMF versus Thymo (P<0.001), FK+AZA versus Thymo (P=0.004), NoCalc versus Thymo (P<0.001);

F Significant differences in UO_8_ between patients receiving CSA+AZA versus FK+MMF (P=0.001), FK+AZA versus FK+MMF (P=0.023), CSA+AZA versus Thymo (P<0.001), CSA+MMF versus Thymo (P=0.002), FK+AZA versus Thymo (P=0.001), NoCalc versus Thymo (P=0.025).

**Table 2:** Correlation between continuous predictors and hourly urine output per kilogram of dry weight in the 1^st^ (UO_1_) and 8^th^ (UO_8_) day after transplantation. N, number and percentages (in relation to the total number, 473) of patients included in analysis; *p*, Spearman's rank correlation coefficient; IQR, interquartile range; UO_1_, hourly urinary output in milliliters per kilogram of dry weight measured between 6 a.m. of postoperative days 1 and 2; UO_8_, hourly urinary output in milliliters per kilogram of dry weight measured between 6 a.m. of postoperative days 8 and 9; CIT, cold ischemia time; Donor creatinine, last measurement of plasma creatinine concentration before donation; PRA, panel of reactive antibodies score; RRT, duration of renal replacement therapy before transplantation in years; CR_1/0_, ratio of plasma creatinine concentration measured in the 1^st^ and 0^th^ postoperative days. *Only 4 patients presented PRA>30%.

	N (%)	Median (IQR)	UO_1_		UO8	
			p	P	p	P
CIT (hours)	444 (93.9%)	17.0 (2.1 - 24.0)	- 0.427	<0.001	-0.369	<0.001
Donor age (years)	451 (96.0%)	35.0 (24.0 - 46.0)	-0.052	0.3	-0.052	0.3
Donor creatinine (mg %)	390 (82.5%)	1.0 (0.9 - 1.2)	-0.321	<0.001	-0.263	<0.001
PRA (%)*	409 (86.5%)	0 (0 - 0)	-0.083	0.09	-0.085	0.08
Patient age (years)	473 (100%)	40.0 (31.5 - 49.5)	-0.165	<0.001	-0.184	<0.001
RRT (years)	447 (94.5%)	3.0 (2.0 - 6.0)	-0.287	<0.001	-0.201	<0.001
CR_1/0_	454 (96.0%)	0.74 (0.5 - 1.0)	–	–	-0.474	<0.001
UO_1_ (mL/Kg h)	473 (100%)	1.9 (1.0 - 2.6)	–	–	0.662	<0.001

CIT inversely correlated with both UO_1_ and UO_8_ (respectively, *p*=-0.43 and −0.37; P<0.001 for both), and CR1/0 inversely correlated with UO_8_ *(p* =-0.47; P<0.001). Patient age inversely correlated with UO_1_ and UO_8_ (p=-0.17 and −0.18; P=0.002 and 0.001, respectively). Urine output was greater in patients receiving kidneys from living-related versus deceased donors (median UO_1_: 2.6 versus 0.6 mL/Kg hour; median UO_8_: 2.2 versus 1.4 mL/ Kg h; P<0.001 for both). UO_1_ and UO_8_ were significantly less in patients that received versus those that did not receive thymoglobulin (median UO1: 0.5 versus 1.3 mL/Kg hour, median UO_8_: 0.5 versus 1.8 mL/Kg hour; P<0.001 for both), and UO_1_ was significantly less in patients that used compared to those that did not use anti-IL2 (0.7 versus 1.3 mL/Kg hour, P=0.02). Urine output progressively decreased with increasing HLA I and II mismatches. Patients with systemic causes of ESRD had significant less UO_8_ than patients with ESRD due to undetermined causes (1.6 versus 2.0 mL/Kg h, P=0.007).

### 

#### Multivariable linear regression

Multiple multivariable linear regressions for both response variables were undertaken with 100 imputed datasets (23). Missing value proportions ranged from 0 to 83 (17.5%, donor creatinine). In the UO_1_ model, we removed organ origin and PRA scores from the predictors’ set as their values were determined from the other predictors (adjusted R^2^ 0.94 and 0.99, respectively) (20, 26). The UO_1_ model initially included nonlinear effects for CIT, RRT and donor creatinine. Stepwise removal of nonlinear effects (donor creatinine then RRT) followed by model comparisons via likelihood ratio tests produced our final model, which admitted nonlinear effects only for CIT.

**Figure 3:**
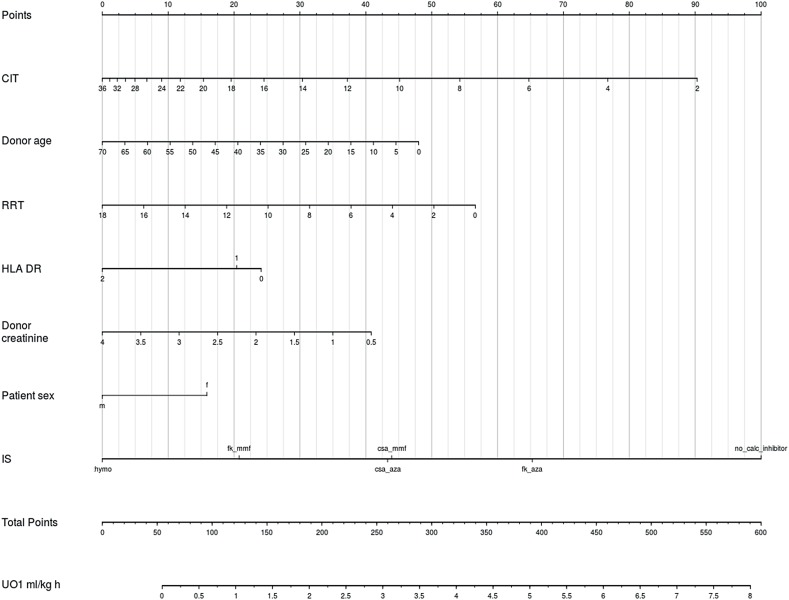
Nomogram to predict UO_8_. In order to obtain the predicted hourly urinary output in mL/Kg, the user identifies each predictors’ values in their respective axes and uses a straightedge to approximate its score in the Points axis. All predictors values are added and this total score is identified in the Total Points axis. The predicted hourly urinary output can then be estimated in the UO_8_ mL/Kg h axis, also with a straightedge. This operation is considerably simplified with the use of a caliper. ESRD etiology: r, renal; s, systemic; u, urologic; a, adpkd; i, undetermined; AR, occurrence of acute cellular rejection within one week of transplantation; Cr_1/0_, ratio between plasma creatinine at postoperative day 1 and 0.

CIT was the most significant predictor of UO_1_ (partial R^2^ 0.067). Initial immunosuppressive regimen, RRT, donor age, patient sex and HLA II compatibility were also able to explain more than 0.5% of UO_1_'s variance (Table-3), and the remaining predictors collectively explained less than 1.7% of UO_1_'s variance. The former variables were included in a nomogram (Figure-3). The mean absolute error of this model was 0.08 mL/Kg hour, and its R^2^ equaled 0.28, decreasing to 0.21 after validation.

**Table 3:** Regression results with partial R2 values from UO_1_ and UO_8_ models. Effects evaluated between the 0.25 and 0.75 percentiles of continuous predictors and between levels of categorical predictors.

Predictor	UO_1_ model			UO_8_ model		
	Effect (CI)	*P* R^2^	P	Effect (CI)	*P* R^2^	P
CIT (2:24)	-1.214 (-1.613 to −0.815)	0.0666	<0.001	-0.090 (-0.306 to 0.126)	0.0007	0.411
Donor age (24:46)	-0.225 (-0.443 to −0.007)	0.0066	0.043	-0.028 (-0.164 to 0.108)	0.0002	0.683
Patient age (31:49)	-0.124 (-0.326 to 0.078)	0.0023	0.228	-0.111 (-0.241 to 0.018)	0.0031	0.092
RRT (2:6)	-0.188 (-0.352 to −0.024)	0.0081	0.025	0.028 (-0.078 to 0.134)	0.0003	0.605
Donor Cr (0.9:1.24)	-0.059 (-0.159 to 0.041)	0.0022	0.246	-0.023 (-0.088 to 0.041)	0.0006	0.479
HLA I mm (3-1:0)	0.121 (-0.368 to 0.611)	0.0047	0.227	-0.067 (-0.361 to 0.226)	0.0011	0.615
HLA I mm (4:0)	0.388 (-0.175 to 0.952)	–	–	0.087 (-0.119 to 0.292)	–	–
HLA II mm (1:0)	-0.056 (-0.381 to 0.270)	0.0053	0.192	0.033 (-0.168 to 0.234)	0.0006	0.744
HLA II mm (2:0)	-0.360 (-0.788 to 0.068)	–	–	0.085 (-0.138 to 0.308)	–	–
Anti-IL2 (1:0)	0.100 (-0.208 to 0.408)	0.0007	0.524	-0.009 (-0.200 to 0.182)	<0.0001	0.926
Patient sex (f:m)	0.237 (-0.013 to 0.487)	0.0056	0.063	0.199 (0.040 to 0.359)	0.0066	0.014
Donor sex (f:m)	0.217 (-0.049 to 0.484)	0.0041	0.110	-0.113 (-0.280 to 0.055)	0.0019	0.188
ESRD (ADPKD:indet)	0.027 (-0.570 to 0.624)	0.0004	0.991	-0.121 (-0.499 to 0.258)	0.0098	0.065
ESRD (renal:indet)	0.001 (-0.329 to 0.331)	–	–	-0.032 (-0.241 to 0.176)	–	–
ESRD (systemic:indet)	-0.004 (-0.300 to 0.292)	–	–	-0.135 (-0.323 to 0.053)	–	–
ESRD (urologic:indet)	0.143 (-0.427 to 0.712)	–	–	0.415 (0.047 to 0.783)	–	–
IS (CSA+AZA:FK+MMF)	0.336 (-0.045 to 0.718)	0.0204	0.027	0.099 (-0.134 to 0.333)	0.0041	0.584
IS (CSA+MMF:FK+MMF)	0.346 (0.006 to 0.686)	–	–	0.099 (-0.115 to 0.312)	–	–
IS (FK+AZA:FK+MMF)	0.665 (0.033 to 1.362)	–	–	0.396 (-0.042 to 0.834)	–	–
IS (NoCalc:FK+MMF)	1.183 (0.205 to 2.161)	–	–	-0.007 (-0.613 to 0.599)	–	–
IS (Thymo:FK+MMF)	-0.310 (-0.874 to 0.253)	–	–	0.016 (-0.332 to 0.365)	–	–
AR	–	–	–	-0.261 (-0.457 to −0.064)	0.0075	0.009
CR_1/0_	–	–	–	-0.118 (-0.300 to 0.064)	0.0018	0.202
UO_1_ (0.3:2.8)	–	–	–	1.155 (0.904 to 1.407)	0.1897	<0.001

CI, 95% confidence interval; ***P*** R^2^, parcial R^2^. CIT, hours of cold Ischemia time; RRT, years in renal replacement therapy before transplantation; HLA I and II, HLA I and II mismatches; Anti-IL2, use of either basiliximab or daclizumab in the initial immunossupressive regimen; ESRD, cause of End-Stage Renal Disease, grouped as systemic, renal, urologic, autossomal dominant polycystic kidney disease (ADPKD) and indeterminate (indet) - see supplemental table 1; IS, immunossupressive regimen: CSA, cyclosporine; AZA, azathioprine; MMF, mycophenolate; NoCalc, no calcineurin inhibitor and no thymoglobulin; Thymo, thymoglobulin; CR_1/0_, ratio of plasma creatinine concentration between the 1^st^ and 0^th^ postoperative days; Donor and patient age in years. Donor creatinine (Donor Cr) in mg%.

**Figure 4:**
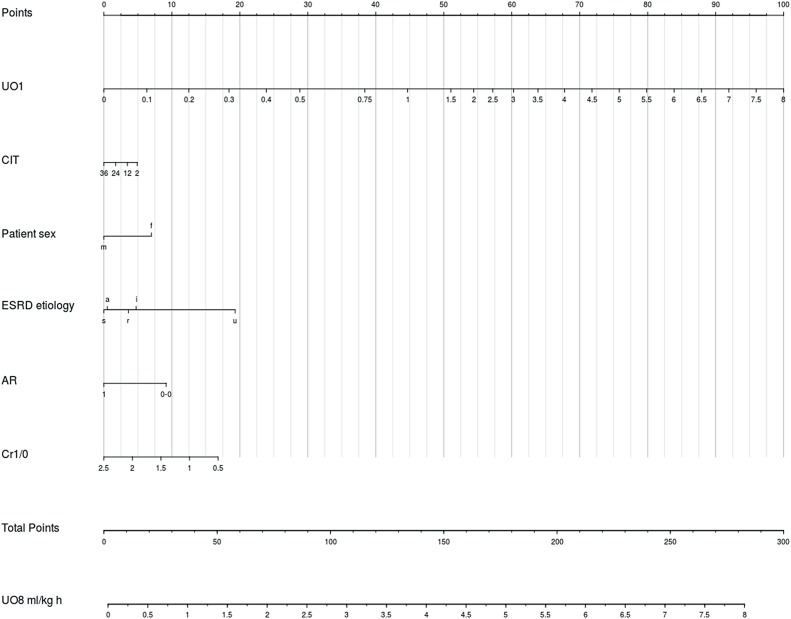
Nomogram to predict UO_8_. In order to obtain the predicted hourly urinary output in mL/Kg, the user identifies each predictors’ values in their respective axes and uses a straightedge to approximate its score in the Points axis. All predictors values are added and this total score is identified in the Total Points axis. The predicted hourly urinary output can then be estimated in the UO_8_ mL/Kg h axis, also with a straightedge. This operation is considerably simplified with the use of a caliper. ESRD etiology: r, renal; s, systemic; u, urologic; a, adpkd; i, undetermined; AR, occurrence of acute cellular rejection within one week of transplantation; Cr_1/0_, ratio between plasma creatinine at postoperative day 1 and 0.

**Supplemental Figure 1 f5:**
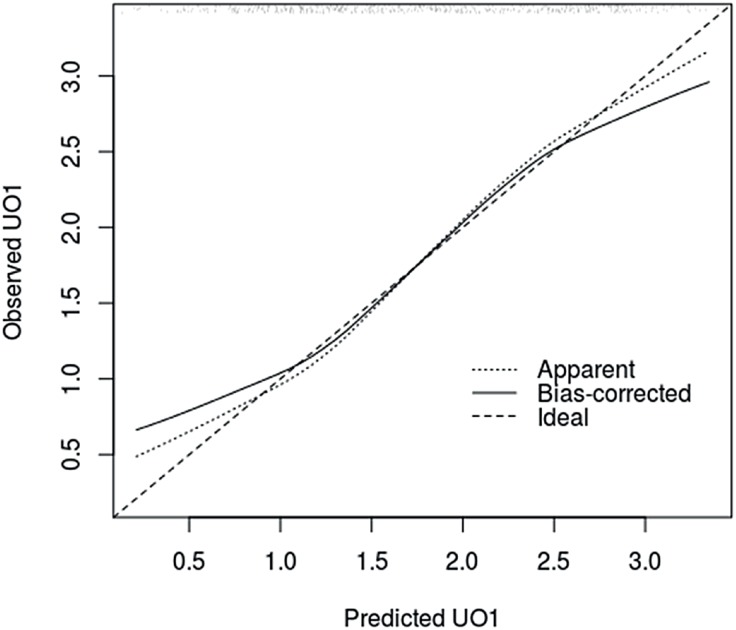
Calibration Plot for the UO_1_ model.

**Supplemental Figure 2 f6:**
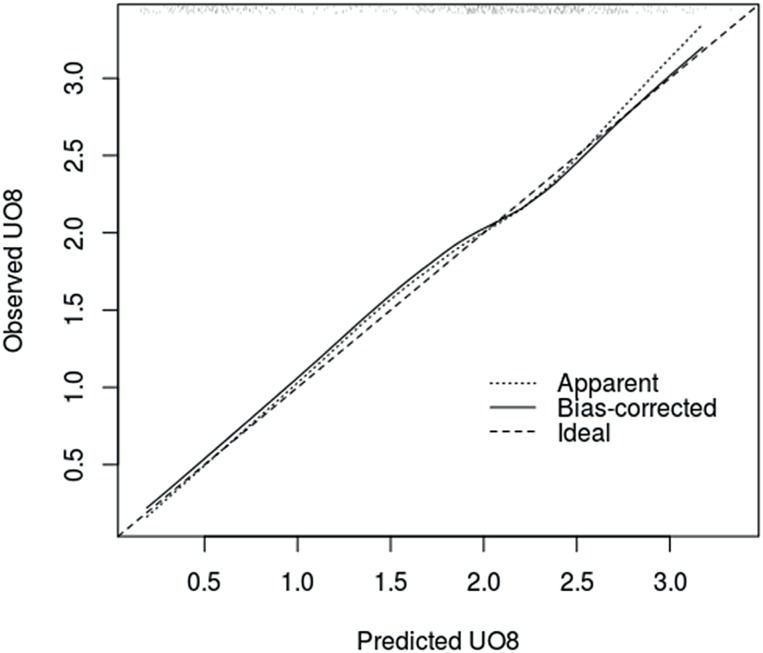
Calibration Plot for the UO_8_ model.

**Supplemental Figure 3 f7:**
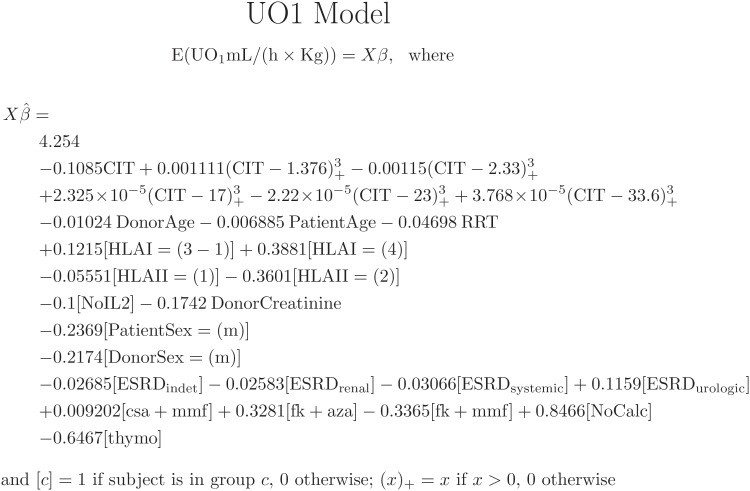
Equation to predict UO_1_.

**Supplemental Figure 4 f8:**
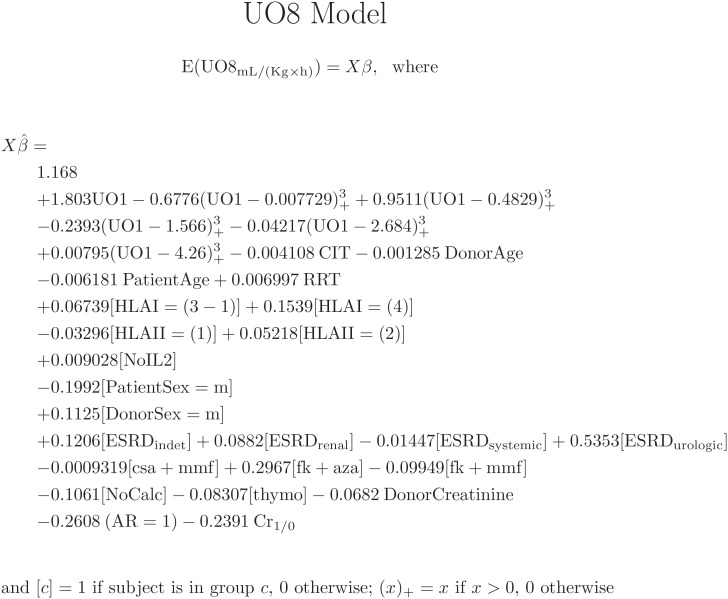
Equation to predict UO_8_.

In the UO_8_ model AR, UO_1_ and Cr_1/0_ were added to the predictor's set. Similar stepwise removal of nonlinear effects and pairwise likelihood tests produced the final model, in which only UO_1_ admitted nonlinear effects. UO_1_ was the strongest predictor of UO_8_ (partial R^2^ 0.19) with AR, ESRD etiology and patient sex also presenting partial R^2≥^0.05. This model's mean absolute error was 0.05 mL/Kg hour, and its 0.53 initial R^2^ was corrected to 0.47 after validation. Besides UO_1_, AR, ESRD etiology and patient sex we included Cr_1/0_ and CIT in a second nomogram for illustrative purposes (Figure-4). Supplemental Figures 1 and 2 depicts the calibration plot for both models. Predictive equations from the UO_1_ and UO_8_ models were also included as supplementary material for examination and external validation purposes (supplemental Figures 3 and 4).

## DISCUSSION

This study indicated CIT as the dominant predictor of early post-transplantation diuresis. Inclusion of early predictors associated with reestablishment of allograft function (UO_1_ and in lesser extent CR_1/0_) and immunologic acceptance of the allograft (AR), almost completely dissipated CIT's ability to predict urine output one week after transplantation. With our regression models we constructed and internally validated nomograms to predict post-transplantation diuresis.

Multiple correlations such as seen in this study benefit from multivariable regression strategies. Yet, interpreting results from these complex models through parameters’ coefficients - which can admit variables’ interactions and nonlinear effects - can challenge even the most proficient analyst. Graphical assessment of these models, in contrast, greatly simplifies the task of understanding these models’ implications. Nomograms are particularly appealing, as they allow straightforward visual appraisal of the contribution of each predictor to the outcome. Nonlinear predictors’ effects, for instance, are depicted with irregular intervals between predictors’ values, which can easily be seen in the CIT axis of our UO_1_ model. The effect of CIT was, nonetheless, monotonically detrimental to UO_1_. Irregular intervals, but with monotonic favorable effects can also be observed in the UO_1_ axis of the UO_8_ model.

Recent years have witnessed the publication of a fair number of nomograms and predictive scores in the field of kidney transplantation, mostly to predict DGF or allograft survival (7, 8, 27). In the study that most resembles ours, as it used a continuous outcome variable, investigators from the Cleveland Clinic (8) developed a nomogram to predict glomerular filtration rate one year postoperatively. Although with merits such a large dataset, absence of variable selection algorithms and allowance for nonlinearity, its considerable number of predictors (18 predictors) may curtail clinical applicability (28). Inclusion of a large set of predictors also marks another study, using the UNOS database (7), which included 11 continuous and 9 categorical variables to predict DGF with moderate-to-high accuracy (c-concordance index =0.704). These studies can be contrasted with the simpler approach offered by Canadian investigators, that developed a nomogram to predict DGF (7) with a much smaller set of predictors: CIT, patient age and weight, HLA-DR mismatches, maximum panel of reactive antibodies (peak PRA) score and donor age. Their leaner predictor's set did not preclude the attainment of a fairly high c-concordance index (0.73).

Indeed, less predictors do render nomograms more transparent and easier to use. A nomogram can be printed on a piece of paper to provide direct visual assessment of how predictors interfere on the specific outcome, thus enabling open discussions with patients and fellow clinicians of the diagnostic and prognostic implications of said predictors (29). On the other hand, a smaller set of predictors can be the limiting element to anticipate multifactorial continuous outcomes, such as early post-transplantation diuresis.

We observed this limitation in predictive ability through the steep increase in optimism-corrected R^2^ between the UO_1_ and UO_8_ models (0.21 to 0.47). This rise in predictive power led us to conjecture that early urine output was most likely influenced by unmeasured variables, and that the aggregate effect of these unmeasured variables was effected through early diuresis (UO_1_), by far the most important predictor of urine output one week after transplantation. Indeed, UO_1_ alone accounted for 35% of the total variance of the UO_8_ model. Corrobatory evidence to this conjecture lies in the fact that CIT, after exerting its key effect on early diuresis, became a remarkably weaker predictor in the UO_8_ model (its partial R^2^ falling from 0.06 to 0.001), for we fail to reason why the effects of these unmeasured variables should not follow an analogous path. Although a large predictors’ set inhibits the clinical use of a predictive tool, one should concede that predicting a continuous variable with truly multivariate causative factors may, ultimately and unavoidably, demand more predictors.

This study has many drawbacks. We are quite aware that urine output is a necessary but not sufficient condition of allograft functional recovery, as metabolic waste products, ions and other molecules must be also be eliminated. We thus acknowledge that our study addresses only one - however fundamental - aspect of kidney function. Also, we did not have access to other potentially important predictors, such as type of harvesting procedure (single versus multiple organ) and preservation solution, patient pre-transplantation diuresis and occurrence of intraoperative hypotension, to name a few. As discussed above, one cannot lightly dismiss the possibility of a significant rise in UO_1_ model's predictive power brought about by these and probably other predictors.

In addition, one may oppose the presence in the dataset of patients receiving both living-related and deceased allografts, considering that brain death has severe autonomic and hemodynamic repercussions that are not wholly encapsulated by CIT. We wanted, however, to assess the widest possible range of CITs, and we hope to have mitigated differences between deceased and living-related allografts by admitting nonlinear CIT effects in the UO_1_ model. Furthermore, we also recognize that one can read our grouping of ESRD causes as arbitrary and therefore a source of classification bias. Still, we consider that any such classification scheme will have some built-in arbitrariness, so that bias may be difficult to avoid. Lastly, we fully acknowledge that our results are conditioned to the peculiarities of our dataset, and advise caution on the part of the reader in the clinical application of our results.

## CONCLUSIONS

This study indicated the preponderant role of CIT in determining early post-transplantation diuresis (UO_1_), with donor age, RRT and choice of initial immunosuppressive regimen playing a secondary - albeit important - role. Urine output one week after transplantation (UO_8_) was mainly determined by early diuresis, and penalized by acute rejection episodes. From these results we developed and internally validated nomograms to predict urine output in the 1^st^ and 8^th^ days after transplantation. The sharp increase in explanatory power between models, however, suggests the existence of preoperative and intraoperative unmeasured variables exerting their effects through early urine output (UO_1_).

We hope that this study inspires other investigators to further explore and improve these predictive models. In particular, which variables may be added in predictive models for early urine output to increase their predictive power. In that we acknowledge that our investigation is but a first attempt to provide the urologic and nephrologic community with what we deem to be an useful predictive tool for the postoperative course of these patients in order to better manage patients’ expectations.

## ABBREVIATIONS

DGF: Delayed Graft Function
SGF: Slow Graft Function
CIT: Cold Ischemia Times
TRIPOD: Transparent Reporting of a Multivariate Prediction Model for Individual Prognosis or Diagnosis
HLA I: Class I (A, B) Human Leucocyte Antigen
HLA II: Class II (DR) Human Leucocyte Antigen
ESRD: End-Stage Renal Disease
anti-IL2: Anti-Interleucin 2 Antibodies
UO_1_: Hourly urine output in postoperative day 1
UO_8_: Hourly urine output in postoperative days 8
**CR**_1/0_: Ratio between plasma creatinine concentration at postoperative days 1 and 0
AR: Biopsy-proven acute cellular rejection
IQR: Interquartile range

## APPENDIX

[Fig f5] [Fig f6] [Fig f7] [Fig f8] [Table t4] [Table t5]

**Supplemental Table 1 t4:** Organ origin according to donor type.

Group	Cause of Brain Death	N (%)
Deceased, vascular		106 (24.4%)
	Subarachnoidal hemorrhage	87 (20.1%)
	Ischemic Stroke	18 (4.1%)
	Acute myocardial infarction	1 (0.2%)
Deceased, non-vascular		176 (40.5%)
	Cranial trauma (blunt)	120 (27.6%)
	Cranial trauma (perforating)	21 (4.8%)
	Brain tumor	18 (4.1%)
	Central hypoxia	13 (3.0%)
	Hypovolemic shock	3 (0.7%)
	Enkephalitis	1 (0.2%)
Living-related donor		152 (35%)
**Total**		**434 (100%)**

**Supplemental Table 2 t5:** Causes of End-Stage Renal Disease.

Aggregated Cause	Cause	N (%)
**Systemic**	–	165 (34.9%)
	Systemic Arterial Hypertension	118 (24.9%)
	Diabetes Mellitus	41 (8.7%)
	Eclampsia	3 (0.6%)
	Hypovolemic Shock	1 (0.2%)
	Thrombotic Microangiopathy	1 (0.2%)
	Septic Shock	1 (0.2%)
**Renal**	–	104 (22.0%)
	Glomerulonephritis (Unspecified)	30 (6.3%)
	Glomerulonephritis (Membranoproliferative)	18 (3.8%)
	Segmental Focal Glomerulonephritis	14 (3.0%)
	Lupus Nephritis	10 (2.1%)
	IgA Nephropathy	9 (1.9%)
	Interstitial Nephritis	8 (1.7%)
	Alport's Disease	7 (1.5%)
	Glomerulonephritis (Rapidly Progressive)	7 (1.5%)
	Glomerulonephritis (Post-Infectious)	1 (0.2%)
**APKD**	–	22 (4.7%)
**Urologic**	–	24 (5.1%)
	Reflux Nephropathy	10 (2.1%)
	Neurogenic Bladder Dysfunction	5 (1.1%)
	Urolithiasis	5 (1.1%)
	Chronic Pyelonephritis (Unspecified)	2 (0.4%)
	Obstructive Uropathy (Unspecified)	1 (0.2%)
	Urethral Stricture	1 (0.2%)
**Indeterminate**	–	158 (33.4%)
**Total**		**473 (100%)**

Causes of End-Stage Renal Disease. APKD, Adult Polycystic Kidney Disease. Percentages rounded to one decimal point.
